# Enhancing Patient Selection in Stage IIIA-IIIB NSCLC: Invasive Lymph Node Restaging after Neoadjuvant Therapy

**DOI:** 10.3390/jcm13020422

**Published:** 2024-01-12

**Authors:** Robert Kwiatkowski, Marcin Zieliński, Jarosław Paluch, Jadwiga Gabor, Andrzej Swinarew

**Affiliations:** 1 Radiotherapy Department, Katowickie Centrum Onkologii, 40-074 Katowice, Poland; 2 Department of Thoracic Surgery, Pulmonary Hospital, 34-500 Zakopane, Poland; zielinski.torako@gmail.com; 3 Department of Laryngology, Faculty of Medical Sciences in Katowice, Medical University Silesia, 40-055 Katowice, Poland; jarek.paluch61@gmail.com; 4 Faculty of Science and Technology, University of Silesia, 75 Pułku Piechoty 1A, 41-500 Chorzów, Poland; 5 Institute of Sport Science, The Jerzy Kukuczka Academy of Physical Education, Mikołowska 72A, 40-065 Katowice, Poland

**Keywords:** Non-Small-Cell Lung Cancer, pneumonectomy, neoadjuvant therapy

## Abstract

Restaging of mediastinal lymph nodes plays a crucial role in the multimodal treatment of stage IIIA Non-Small-Cell Lung Cancer (NSCLC). This study aimed to assess the impact of restaging using endobronchial ultrasound (EBUS), endoesophageal ultrasound (EUS), and transcervical extended mediastinal lymphadenectomy (TEMLA) after neoadjuvant chemotherapy (CHT) or chemoradiotherapy (CRT) on the 5-year overall survival (OS) of patients with NSCLC diagnosed with clinical stage IIIA-IIIB and metastatic ipsilateral mediastinal nodes (N2) who underwent radical pulmonary resections. Patients diagnosed with stage IIIA-IIIB NSCLC and N2 mediastinal nodes were included in this study. Restaging of mediastinal lymph nodes was performed using EBUS, EUS, and TEMLA. The patients were divided into two groups based on the restaging method: the TEMLA restaging group and the chest CT scan-only group. The primary outcome measure was the 5-year OS rate, and the secondary outcome measures included median OS and survival percentages. Statistical analysis, including the log-rank test, was conducted to assess the differences between the two groups. The TEMLA restaging group demonstrated significantly better overall survival compared to the chest CT scan-only group (log-rank test, *p* = 0.02). This was evident through a four-fold increase in median OS (59 vs. 14 months) and a higher 5-year OS rate of 55.9% (95% CI: 40.6–71.1) compared to 25.0% (95% CI: 13.7–36.3) in the chest CT scan-only group (*p* = 0.003). Invasive restaging of mediastinal lymph nodes improves the selection of patients with stage IIIA-IIIB (N2) NSCLC after neoadjuvant therapy. The use of EBUS, EUS, and TEMLA provides valuable information for identifying patients who may benefit from surgery by identifying N2 to N0-1 downstaging. These findings emphasize the importance of incorporating restaging procedures into the treatment decision-making process for NSCLC patients with mediastinal lymph node involvement.

## 1. Introduction

Lung cancer continues to be a significant global health challenge, claiming more lives than any other type of cancer. In 2013 alone, there were an estimated 1.8 million new cases and 1.6 million deaths attributed to this devastating disease, accounting for approximately 19% of all cancer-related deaths [1,2]. Despite advancements in treatment options, the overall survival rate for lung cancer remains dishearteningly low, with only 16.3% of patients surviving beyond five years following diagnosis [3].

Efficient and personalized treatment strategies are crucial for maximizing the chances of a cure for early-stage lung cancer and avoiding unnecessary invasive procedures in advanced cases. Thus, accurate TNM staging plays a pivotal role in determining the most appropriate course of action for patients with Non-Small Cell Lung Cancer (NSCLC) [4].

Among the NSCLC patient population, those diagnosed with stage IIIA NSCLC and harboring metastatic ipsilateral mediastinal nodes (N2) represent a unique and highly debated subgroup. Although initial surgical intervention is not recommended for this particular group, the possibility of radical surgery after neoadjuvant therapy and subsequent restaging offers a glimmer of hope [5,6,7].

Various methods are employed for the staging of NSCLC, encompassing a range of imaging techniques, such as chest CT, PET-CT, and endoscopic/ultrasound approaches, such as endobronchial ultrasound/transbronchial needle aspiration (EBUS/TBNA) and endoscopic ultrasound/fine needle aspiration (EUS/FNA). Surgical techniques, including standard cervical mediastinoscopy (CM), video-assisted mediastinoscopy (VAM), extended mediastinoscopy, video-assisted mediastinoscopic lymphadenectomy (VAMLA), transcervical extended mediastinal lymphadenectomy (TEMLA), anterior mediastinotomy (Chamberlain procedure), and video thoracoscopy (VATS), are also employed [8,9,10]. Presently, EBUS/TBNA and EUS/FNA are considered the next phase of mediastinal staging following CT and PET/CT, providing a comprehensive assessment of the mediastinal nodes [11,12,13]. EBUS and EUS are complementary techniques that allow for visualization and biopsy of most of the mediastinal nodes [14]. Notably, TEMLA, a relatively novel technique involving the complete removal of the mediastinal nodes and surrounding adipose tissue, aims to enhance the accuracy of NSCLC staging and restaging after neoadjuvant treatment [10,15,16].

Restaging of mediastinal nodes holds significant importance in the context of multimodality treatment for stage IIIA NSCLC. Several studies have demonstrated that patients with residual metastatic nodes after neoadjuvant therapy exhibit inferior survival rates compared to those with nodal downstaging to ypN0-1 [10,17]. This disparity becomes more pronounced in patients with residual multilevel metastatic nodes [18,19]. Consequently, the decision to pursue surgery after neoadjuvant therapy should be based on the most reliable restaging method available. Currently, several approaches are available to restage mediastinal lymph nodes following neoadjuvant treatment, including imaging studies such as chest CT, PET/CT, EBUS, EUS, and combined EBUS/EUS, as well as invasive procedures such as repeated mediastinoscopy (re-mediastinoscopy), VATS, and TEMLA.

The objective of this study was to analyze the impact of restaging the mediastinal lymph nodes using endobronchial ultrasound (EBUS), endoesophageal ultrasound (EUS), and transcervical extended mediastinal lymphadenectomy (TEMLA) after neoadjuvant chemotherapy (CHT) or chemoradiotherapy (CRT) on the five-year overall survival of patients diagnosed with Non-Small Cell Lung Cancer (NSCLC) in clinical stage IIIA-IIIB with metastatic ipsilateral mediastinal nodes (N2) undergoing radical pulmonary resections.

## 2. Materials and Methods

This retrospective, single-center analysis included two groups of NSCLC patients diagnosed with stage IIIA disease based on CT scans that revealed enlargement of the ipsilateral mediastinal and/or subcarinal lymph nodes (N2) that responded to neoadjuvant chemotherapy (CTH) or chemoradiotherapy (CRT) and underwent surgery after restaging.

The first group (N = 56) was treated between 1999 and 2005 with restaging based exclusively on chest CT scans (clinical restaging group).

**Figure 1:**
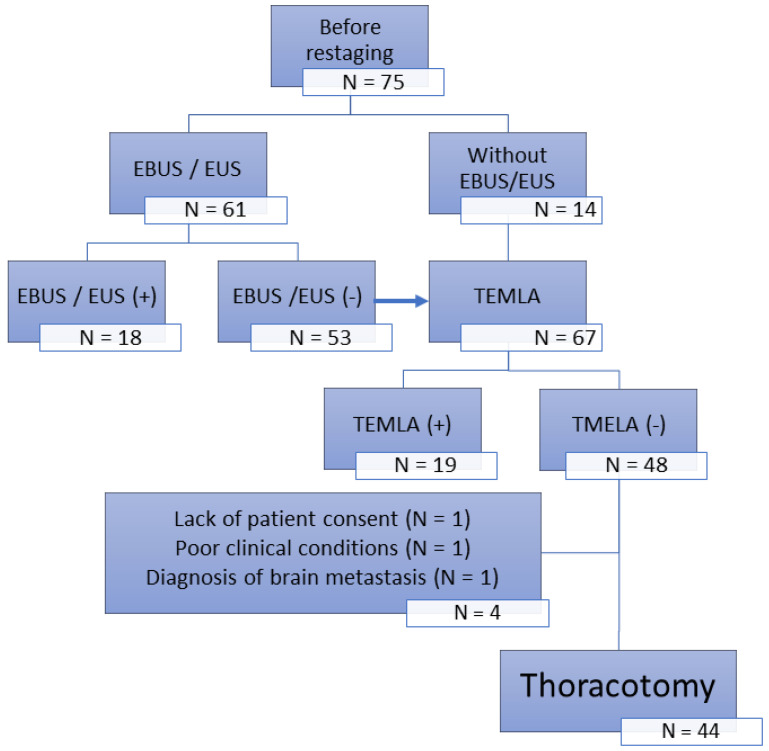
Diagram of diagnostic procedures for invasive N2 lymph nodes restaging in the group of patients with Non-Small-Cell Lung Cancer (NSCLC) diagnosed in clinical stage IIIA-IIIB with metastatic ipsilateral mediastinal nodes (N2) after neoadjuvant therapy.

The second group (N = 75) was treated from 2004 to 2008 with restaging based on CT scans with verification of mediastinal lymph nodes by transcervical extended mediastinal lymphadenectomy (TEMLA) performed in 2006 by endobronchial ultrasound (EBUS)/endoesophageal ultrasound (EUS). The final analysis included 44 operated patients without lymph node involvement confirmed by TEMLA (TEMLA restaging group; Figure 1).

### 2.1. Outcomes

The analysis of outcomes included percentages of exploratory thoracotomies, in-hospital postoperative mortality, overall survival (OS), and 5-year overall survival (5-year OS) in the clinical and TEMLA restaging groups.

### 2.2. Statistical Analysis

The statistical analysis was performed using STATISTICA 13.3 PL (13.3.0. TIBCO Software Inc., Palo Alto, CA, USA). The data are presented as mean values with standard deviations. The normality distribution was verified using the Shapiro–Wilk test. The Students’ *t*-test was used to compare differences in means. The statistical analysis also included the independence test c2, using which the two groups were compared in terms of sex, histologic type of cancer, type of neoadjuvant therapy, type of surgery performed, and clinical stage.

Differences in the Kaplan–Meier survival curves between the groups were calculated using the log-rank test. In addition, the median OS with a 95% confidence interval was calculated. For all analyses, a *p*-value below 0.05 was considered statistically significant.

## 3. Results

### 3.1. Analyzed Group Characteristics’

Squamous cell carcinoma was the predominant NSCLC type in both study groups (Table 1). There were significant differences in adjuvant therapy. In the non-invasive restaging group, CRT was the most frequent method (80.4%), while in the TEMLA restaging group, it was CTH (86.4%). There was no difference between the study groups regarding age, sex, histologic type of tumor, type of surgical procedure, and resection margins.

### 3.2. Overall Survival

**Figure 2:**
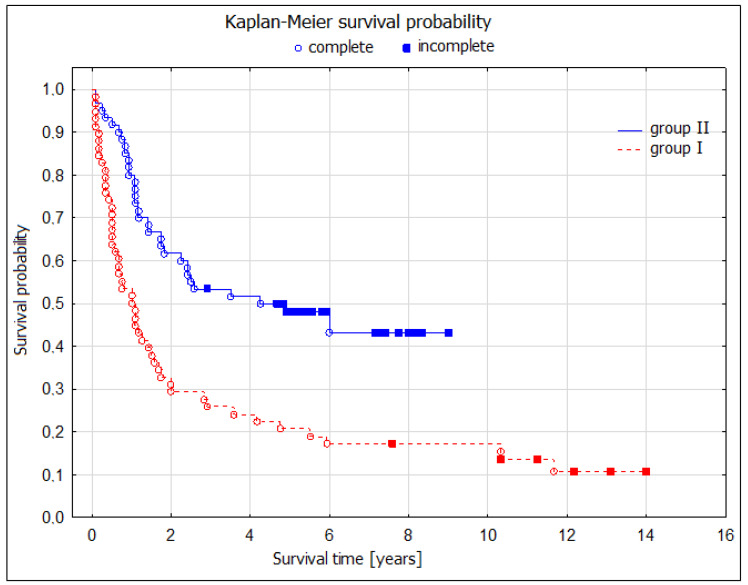
Five-year overall survival (OS) in patients with Non-Small-Cell Lung Cancer (NSCLC) diagnosed in clinical stage IIIA-IIIB with metastatic ipsilateral mediastinal nodes (N2), undergoing radical pulmonary resections after neoadjuvant therapy and N2 nodes restaging based on CT scans (Non-invasive restaging group-group I) or transcervical extended mediastinal lymphadenectomy (TEMLA group- group II).

During the 5-year postoperative period, there were 42 deaths (75%) in the noninvasive restaging group and 20 deaths (45.5%) in the TEMLA restaging group. These numbers were reflected by significantly better 5-year overall survival (OS) after TEMLA restaging: 55.9 (95% CI: 40.6–71.1) vs. 25.0 (13.7–36.3)%, *p* = 0.003; four times longer median OS (59 vs. 14 months); log-rank test, *p* = 0.02 (Figure 2).

We also obtained similar results and significant differences in 5-year OS when we analyzed separately men [58.1 (CI: 41.8–74.3) vs. 20.0 (CI: 8.3–31.7)%; *p* < 0.001], patients in the age of less than 60 years old [42.9 (6.2–79.5) vs. 17.6 (0.1–35.8)%, *p* = 0.01)], those with squamous cell carcinoma [56.1 (38.8–73.3) vs. 29.4 (14.1–44.7)%, *p* = 0.009], after pneumonectomy [60.0 (38.5–81.5) vs. 28.0 (10.4–45.6)%, *p* = 0.03], and R0 resections [55.9 (40.6–71.1) vs. 25.0 (12.8–37.3)%, *p* < 0.001]. While there was no difference among women (*p* = 0.21), patients aged 60 years or older (*p* = 0.15), with adenocarcinoma (*p* = 0.18), and non-other specified histology—NOS (*p* = 0.09), after lobectomy (*p* = 0.06), and R1-2 resections (*p* = 0.78), these results were compiled and are presented in the table below (Table 2).

There was also no difference in the 5-year OS between the subgroups of patients with different pathologic responses to neoadjuvant CTH/CRT (yp) (Table 3).

## 4. Discussion

Current guidelines for the treatment of patients with stage IIIA (N2) Non-Small Cell Lung Cancer (NSCLC) are primarily based on the findings of two major prospective randomized trials conducted by the RTOG and the EORTC [20,21]. These trials concluded that concurrent chemoradiotherapy (CRT) is the preferred treatment method because it is less invasive than surgery. However, a more detailed analysis of the trial results revealed that the presence of persistent metastases in N2 lymph nodes had a significantly negative impact on survival outcomes. Interestingly, in the subgroup of patients who showed downstaging from N2 to N1/0 after neoadjuvant treatment, those treated with surgery demonstrated significantly better 5-year overall survival (OS) rates than those treated with CRT. This interpretation suggests that surgical intervention should be limited to patients who achieve remission of mediastinal node metastases (downstaging from N2 to N0/1) after neoadjuvant therapy. However, such selection can only be reliably determined through highly sensitive and specific restaging techniques, which were not utilized in the RTOG and EORTC studies that relied solely on chest CT for restaging purposes [5,6,7,19,22,23,24,25].

In our single-center retrospective analysis, we investigated two groups of patients with stage III NSCLC and ipsilateral mediastinal and/or subcarinal lymph node metastases (N2). Restaging was performed using different methods: chest CT alone in the group treated between 1999 and 2003 and the introduction of endobronchial ultrasound (EBUS), endoscopic ultrasound (EUS), and transcervical extended mediastinal lymphadenectomy (TEMLA) in the latter group (2004–2008). The transitional period from 2004 to 2005 involved some patients who qualified for treatment based on both scenarios. Notably, PET-CT was not utilized in either group because of its limited availability at the time.

The TEMLA restaging group demonstrated significantly better overall survival compared to the chest CT scan-only group (log-rank test, *p* = 0.02). This was evident through a four-fold increase in median OS (59 vs. 14 months) and a higher 5-year OS rate of 55.9% (95% CI: 40.6–71.1) compared to 25.0% (95% CI: 13.7–36.3) in the chest CT scan-only group (*p* = 0.003). The analysis revealed a significantly superior 5-year OS in patients who underwent TEMLA-based restaging and subsequently qualified for pulmonary resection (Figure 2).

This difference was particularly pronounced in pneumonectomy cases, as lobectomy did not demonstrate statistical significance. These findings contradict those reported by Albain and Van Meerbeck [20,21]. In our opinion, poor results from pneumonectomy were probably due to technical problems or the lack of experience of the surgeons who performed these procedures. In the other publications on the role of pneumonectomy, 90-day mortality was 2–4%. There is no doubt that pneumonectomy is a demanding procedure for patients; however, this option should be considered if this is the only way to achieve a curative resection [26,27,28].

Surprisingly, we observed a higher 5-year OS among patients who underwent more extensive surgery, namely pneumonectomy, than among those who underwent lobectomy. Although the difference was not statistically significant, it suggests the value of pneumonectomy in the treatment of patients with NSCLC following neoadjuvant therapy.

Further analysis revealed that the statistically significant superiority of TEMLA restaging was notable among younger patients (<60 years) with squamous cell carcinoma and R0 resection. However, no survival benefit was observed in women, patients over 60 years old, those who underwent non-radical operations (R1-2), and other histological types such as adenocarcinoma and not otherwise specified (NOS). It is important to note that these subgroups were small, rendering the analysis underpowered. Nevertheless, the lack of a survival benefit in non-radically operated patients (R1-2) underscores the importance of maximizing preoperative imaging with available methods to minimize the selection of patients for surgical treatment that would not derive significant benefits. In our opinion, TEMLA is one such method that can contribute to this goal. Based on our findings, we propose that surgical treatment for stage IIIA-IIIB (N2) NSCLC should only be offered to patients who demonstrate downstaging of ipsilateral metastatic mediastinal lymph nodes following neoadjuvant therapy, while it should be avoided in cases without nodular regression.

Another intriguing observation was the marked difference in 5-year OS between patients who underwent noninvasive restaging and TEMLA restaging with R0 radical resections. This difference (25.0% vs. 55.9%) suggests a potential therapeutic effect of bilateral extended lymphadenectomy (TEMLA). However, as chest CT was more frequently used in the TEMLA restaging group, this association could not be definitively proven. This observation underscores the need for further studies to explore potential therapeutic effects.

In conclusion, invasive mediastinal lymph node restaging improves the selection of patients with stage IIIA-IIIB (N2) NSCLC after neoadjuvant therapy who would benefit from a multidisciplinary treatment approach, including surgery. Our study highlights the importance of employing more advanced restaging techniques, such as TEMLA, to accurately assess the response to neoadjuvant therapy and guide appropriate treatment decisions. Further research is necessary to confirm these findings and to elucidate the potential therapeutic benefits of bilateral extended lymphadenectomy in this patient population.

## 5. Conclusions

Invasive mediastinal lymph node restaging, specifically through techniques such as TEMLA, improves the selection of patients with stage IIIA-IIIB (N2) NSCLC who would benefit from a multidisciplinary treatment approach, including surgery after neoadjuvant therapy.

Patients who demonstrate downstaging of ipsilateral metastatic mediastinal lymph nodes following neoadjuvant therapy are more likely to have better 5-year overall survival rates when undergoing surgical intervention.

Further research is needed to confirm these findings and explore the potential therapeutic benefits of bilateral extended lymphadenectomy (TEMLA) in this specific patient population.

Only patients who benefited from induction treatment, downstaging in the area of mediastinal lymph nodes, benefited from surgical treatment.

## Figures and Tables

**Table 1:** Characteristics of patients with Non-Small-Cell Lung Cancer (NSCLC) diagnosed in clinical stage IIIA-IIIB with metastatic ipsilateral mediastinal nodes (N2), undergoing radical pulmonary resections after neoadjuvant therapy, and N2 nodes restaging based on CT scans (Non-invasive restaging group) or transcervical extended mediastinal lymphadenectomy—(TEMLA).

	Non-Invasive Restaging (Group I) [N = 56]	TEMLA Restaging (Group II) [N = 44]	Statistical Significance
Gender [N]			
Women	11	8	0.94
Men	45	36	
Age [years]	56 ± 9	58 ± 7	0.13
Histological type [N]			
Adenocarcinoma	17	7	0.24
Squamous cell	34	32	
Other/NOS	5	5	
Neoadiuvant therapy [N]			
Chemotherapy	11	38	<0.001
Chemoradiotherapy	45	6	
Type of surgery [N]			
Pneumonectomy	25	20	0.73
Lobectomy	28	23	
Thoracotomy	3	1	
Resection margins [N]			
R0	43	40	0.39
R1/2	10	3	

**Table 2:** Five-year overall survival (OS) according to gender, histology, the method of neoadjuvant treatment, type of surgery, and resection margins (R0-2) of patients with Non-Small-Cell Lung Cancer (NSCLC) diagnosed in clinical stage IIIA-IIIB with metastatic ipsilateral mediastinal nodes (N2), undergoing radical pulmonary resections after neoadjuvant therapy, and N2 nodes restaging based on CT scans (Non-invasive restaging group) or transcervical extended mediastinal lymphadenectomy (TEMLA).

	Non-Invasive Restaging (Group I) [N = 56]		TEMLA Restaging (Group II) [N = 44]	
	N	5-Year Survival N (%)	N	5-Year Survival N (%)
Total	56	14 (25)	44	24 (54.5)
Gender				
Women	11	3(27.3)	8	3 (37.5)
Men	45	11 (24.4)	36	21 (58.3)
Histological type				
Adenocarcinoma	17	3 (17.6)	7	3 (42.8)
Squamous cell	34	10 (29.4)	32	18 (56.2)
Other/NOS	5	1 (20)	5	3 (60)
Neoadjuvant therapy				
Chemotherapy	11	2 (18.2)	38	18 (47.4)
Chemoradiotherapy	45	12 (26.7)	6	6 (100)
Type of surgery				
Pneumonectomy	25	7 (28)	20	12 (60)
Lobectomy	28	7(25)	23	11 (47.8)
Thoracotomy	3	0	1	1(100)
Resection margins				
R0	43	12 (27.9)	40	22 (55)
R1/2	10	2 (20)	3	1 (33.3)

**Table 3:** Five-year overall survival (OS) according to pathological response to neoadjuvant therapy (yp) in patients with Non-Small-Cell Lung Cancer (NSCLC) diagnosed in clinical stage IIIA-IIIB with metastatic ipsilateral mediastinal nodes (N2), undergoing radical pulmonary resections after N2 nodes restaging based on CT scans (Non-invasive restaging group), or transcervical extended mediastinal lymphadenectomy (TEMLA).

	Non-Invasive Restaging (Group I) [N = 56]		TEMLA Restaging (Group II) [N = 44]	
	N	5-Year Survival [N (%)]	N	5-Year Survival [N (%)]
yp0	10	5 (50)	3	2 (67)
ypI	21	5 (24)	23	11 (48)
ypII	8	2 (25)	8	5 (62)
ypIIIa	14	2 (14)	9	5 (56)
ypIIIb	3	0	1	1 (100)

## Data Availability

All data are contained within this article.
